# Genotype and phenotype spectrum of 10 children with *STXBP1* gene-related encephalopathy and epilepsy

**DOI:** 10.3389/fped.2022.1010886

**Published:** 2022-11-11

**Authors:** Meng Dong, Tianyu Zhang, Ruimei Hu, Meng Li, Guan Wang, Xinjie Liu

**Affiliations:** Department of Pediatrics, Qilu Hospital, Shandong University, Jinan, China

**Keywords:** *STXBP1* mutations, early infantile epileptic encephalopathy, intellectual disability, phenotype spectrum treatment, prognosis

## Abstract

**Objective:**

*STXBP1* mutations are associated with early onset epileptic encephalopathy (EOEE). Our aim was to explore the phenotype spectrum, clinical treatment and prognosis of *STXBP1*-related encephalopathy (*STXBP1*-E).

**Methods:**

Clinical and genetic data were collected from 10 patients with *STXBP1* mutations. These patients were examined and diagnosed from 2015 to 2021 at the Pediatric Department of Qilu Hospital. Blood samples were collected and sequenced by next generation sequencing and Candidate pathogenic variants were identified using Sanger sequencing in all family members.

**Results:**

All of the patients showed severe epilepsy, varying degrees of intellectual disability and delayed motor. The patients developed multiple seizure types and abnormal electroencephalography (EEG) results at onset, and focal seizures were the most frequent seizure type. Among the patients, 2 were diagnosed with Ohtahara syndrome, 2 patient was diagnosed with West syndrome. The other 6 patients could not be diagnosed with any specifically recognized epilepsy syndrome. Five of the 10 patients had a history of fever with seizures, 4 of whom had eliminated intracranial infection according to the results of cerebrospinal fluid (CSF) examinations, and the other patient was diagnosed with anti-myelin oligodendrocyte glycoprotein (MOG) -associated encephalitis. We identified one patient with a complete deletion of *STXBP1* and 9 patients with *de novo* heterozygous mutations of *STXBP1*. Among those mutations, 4 were novel (c.56°C > T, c.1315A > T, c.751G > C, and c.554_559del), and 5 had been previously reported [c.364C > T, c.569G > A (2 cases), c.748C > T, and c.1651C > T]. For 8 of our patients, different combinations of anti-seizure medications (ASMs) led to seizure freedom. One patient with MOG antibodies in his serum obtained a poor therapeutic effect from the traditional ASMs treatment, so he had to achieve seizure-free status through vagus nerve stimulation (VNS), which had little effect on his psychomotor ability. Fortunately, in one case, patient psychomotor ability was improved through VNS.

**Conclusion:**

Our study shows that *STXBP1* screening should be considered in patients with neonatal seizures with intellectual disability, and frequent seizures with fever should also be considered with the *STXBP1* mutation when intracranial infection is eliminated. VNS has expanded outcome measures to include behavioral and developmental function as well as seizure control.

## Introduction

Early-onset epileptic encephalopathy (EOEE) embodies the notion that continuous and repeated epileptic activity itself may contribute to severe neurological and cognitive impairment and prominent interictal epileptiform discharges during the neonatal or early infant period (1). After the initial identification of mutations for cryptogenic West syndrome and Ohtahara syndrome (2), in the genes *ARX, CDKL5, STXBP1, SLC25A22, SPTAN1, PLCb1, MAGI2, PNKP, SCN1A*, numerous other mutated genes for West syndrome, Dravet syndrome and various other types of childhood-onset epilepsy have been found, revealing that a significant proportion of cryptogenic EOEEs are single-gene disorders (3–5). West syndrome, also known as epileptic spasms or infantile spasms which belong to infantile epileptic spasms syndrome (IESS) (6). In 2008, the first mutation in the syntaxin binding protein 1 (*STXBP1*) gene was confirmed to be associated with EOEE (7). Since then, mutations in *STXBP1* have been described in different patient cohorts, the phenotypic spectrum of patients with *STXBP1* mutations has expanded, and an increasing number of studies have indicated that epilepsy and intellectual disease (ID) are 2 major features of *STXBP1* encephalopathy (*STXBP1-E*) (7, 8). However, Hamdan et al. reported a *STXBP1*-positive patient with mild nonsyndromic ID without epilepsy (9). Presently, *STXBP1-E* is considered a complex neurodevelopmental disorder rather than a primary epileptic encephalopathy (10). Previous studies have reported that vigabatrin, valproic acid (VPA), levetiracetam (LEV), adrenocorticotropic hormone and a ketogenic diet (KD) are effective at seizure control (11–15). However, the prognosis of the child is not good, so it is necessary to deeply understand this disease and improve the prognosis. Our study not only expands the phenotypic spectrum associated with *STXBP1-E* but also describes the role of VNS in controlling epilepsy and improving cognition.

## Materials and methods

### Clinical data and phenotypes groups

All the patients diagnosed EOEE were detected by next-generation sequencing from Jan 2015 to Jul 2021 at the Pediatric Department in Qilu Hospital and only ten previously unreported patients with a *STXBP1* mutation were enrolled in this study. Clinical data [classification in epileptic syndrome, epileptic spasms, ambulatory or video EEG monitoring, magnetic resonance imaging (MRI), history of status epilepticus, treatments, and follow-up until July 2021] were collected from their clinical cases. Approximately 2 ml of peripheral blood (plus EDTA anticoagulant) was obtained from the patient and their parents after receiving written informed consent.

We refer to the study from Wolking et al. (16), they discerned 4 different phenotypic groups and divided into four groups [developmental and epileptic encephalopathies, DEE; genetic epilepsies with febrile seizures (FS) plus, GEFS+; genetic generalized epilepsy, GGE; focal epilepsy] according to their clinical characters.

The investigation was performed with the approval of the ethics committee of Qilu Hospital.

### Genetic analysis

Genomic DNA was extracted using a QIAamp Blood Midi Kit (QIAGEN, Valencia, CA). To identify disease-causing gene variants, a GenCap panel with 175 genes (including *STXBP1*, Table 1) associated with epilepsy was customized, and a capture strategy was performed using the GenCap custom enrichment kit (MyGenostics Inc, Beijing, China). An Illumina NextSeq 500 sequencer (Illumina, San Diego, CA, United States) was used with 150 bp paired-end reads. An ABI3730xl sequencer (Applied Biosystems, United States) was used to apply the Sanger sequencing method, and the results were compared to the capture sequencing results.

**Table 1:** Detected gene list.

PAH	L2HGDH	DHTKD1	CTH	ARX	MMACHC	ATP7B	GCSH
PTS	D2HGDH	GALK1	MTHFR	SLC6A8	MMAA	G6PD	CPS1
GCH1	IDH2	INPP5E	MTRR	GAMT	MMAB	ATP7A	OTC
QDPR	ETFA	LAMP2	MTR	GATM	ABCD4	PTPN11	ASS1
PCBD1	ETFB	MAOA	GNMT	ERCC8	GPHN	MVK	ASL
SPR	ETFDH	PNPLA2	AHCY	ERCC6	MCEE	ACSF3	ARG1
FAH	BCAT1	SLC2A1	SLC25A13	SLC46A1	MMADHC	OGDH	OAT
TAT	BCAT2	HSD17B10	SLC25A15	SLC19A1	HCFC1	FH	NAGS
HPD	SLC22A5	SLC2A2	GLUD1	FOLR1	LMBRD1	AASS	SLC7A7
HGD	CPT1A	SLC3A1	GLUL	FOLR2	GCDH	ABHD5	MAT1A
HAL	CPT2	SLC7A9	BCKDHA	DHFR	HMGCL	ACAT1	CBS
UROC1	SLC25A20	GLYCTK	BCKDHB	DDC	AUH	ADK	SUOX
FTCD	MLYCD	TYMP	DBT	PHGDH	TAZ	ALDH6A1	MOCS1
GLDC	ACADSB	TK2	DLD	PSAT1	OPA3	ASPA	MOCS2
AMT	ACADS	DGUOK	SARDH	ABAT	SERAC1	DBH	NR0B1
ACADM	POLG	PRODH	ALDH5A1	ALDH7A1	FBXL4	MCCC2	SOX9
ACADVL	SUCLA2	ALDH4A1	SRY	ALPL	KMT2D	PCCA	CYP21A2
HADHA	MPV17	SLC6A20	AR	PNPO	KDM6A	PCCB	CYP11B1
HADHB	C10orf2	SLC6A19	HSD17B3	ETHE1	SGSH	HLCS	HSD3B2
HADH	RRM2B	SLC36A2	SRD5A2	FOXG1	NAGLU	BTD	CYP17A1
ACAD8	SUCLG1	IVD	NR5A1	MECP2	HGSNAT	PC	StAR
TH	SLC25A4	MCCC1	WT1	CDKL5	GNS	MUT	

After sequencing, the raw data were saved in FASTQ format. Quality control (QC) filters were applied to remove reads with low quality. Then, the clean reads were assembled and spliced using the second-generation sequencing analysis platform provided by MyGenostics and the coverage and sequencing quality of the target region were evaluated. Finally, flash analysis platform was used to analyze the pathogenicity of variation, and the possible variation loci were determined. The pathogenicity of variation loci was also analyzed according to ACMG (American College of Medical Genetics and Genomics) genetic variation classification criteria and guidelines.

We performed a conservative analysis of the eight mutant amino acid sequences using Clustal Omega. Domains of *STXBP1* were identified based on the National Center for Biotechnology Information (NCBI) Conserved Domain Database. Multiple sequence alignment of *STXBP1* was performed using the ClustalW program. Three-dimensional structural models of *STXBP1* were predicted with the Swiss-model web tool. Protein structure images were generated using the PDB file and PyMOL. Hydrogen bonds in the proteins were demonstrated using PyMOL to predict changes in mutant stability.

## Results

### *STXBP1* molecular analysis

**Figure 1:**
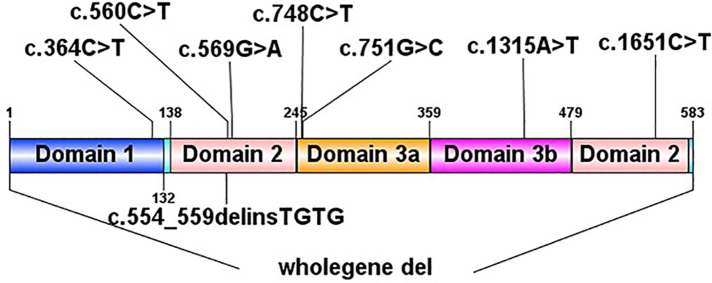
Pathogenic STXBP1 mutations.

In total, we identified 9 *de novo* STXBP1 mutations in 10 patients by next generation sequencing to sequence all the *STXBP1* exons and splice junction boundaries from the genomic DNA. Upon searching in the HGMD (The Human Gene Mutation Database), 6 of them had been reported previously [c.364C > T, c.569G > A (2 cases), c.748C > T, c.1651C > T, c.56°C > T, one complete deletion], and the other 3 were novel mutations (c.1315A > T, c.751G > C, c.554_559del). Two unrelated patients (patient No. 2 and patient No. 8) had the same *STXBP1* nucleotide alteration (c.569G > A). The details of the *STXBP1* mutations are summarized in Table 2 and Figure 1. According to ACMG (American College of Medical Genetics and Genomics) criteria, we have determined that the variants are Pathogenic variants (Supplementary Figures S1, S2).

**Table 2:** Summary of the variants in the STXBP-1 mutations.

Patients	Exon	Nucleotide	Protein	Pathogenic analysis	Reference
1	exon6	c.364C > T	p. R122X	pathogenic	(17, 18)
2	exon7	c.569G > A	p. R190Q	pathogenic	(19)
3	exon7	c.56°C > T	p. P187l	uncertain	(20)
4	exon9	c.748C > T	p. Q250X	Pathogenic	(17)
5	exon15	c.1315A > T	p. I439F	uncertain	novel
6	exon18	c.1651C > T	p. R551C	Pathogenic	(21)
7	exon9	c.751G > C	p. A251P	Likely pathogenic	novel
8	exon7	c.569G > A	p. R190Q	Pathogenic	(19)
9	wholegene	c150928c02101	–	Pathogenic	(22)
10	exon7	c.554_559delinsTGTG	p. E185fs*28	uncertain	novel

### Clinical characterization of *STXBP1*-positive of the 10 patients

The clinical features of this study with EOEE having *STXBP1* defects are summarized in Table 3. The median age of ascertainment was 14.5 months (*n* = 10, range = 1–35 months), with a median age of seizure onset of 8.5 months (range = 0–22 months), and most patients had intellectual disability prior to seizure onset (60%). At the last follow-up, all patients had development delay. Patient 1 and patient 2 were diagnosed with Ohtahara syndrome, and patient 1 developed into West syndrome at the age of 5 months. Two patients had West syndrome (patient 4, 9), and 6 patients were diagnosed with unclassified EOEEs (patients 2, 3, 5, 6, 7, and 8). Various seizure types presented during the course of the disease, including epileptic spasms, partial seizures, atonic seizures, absence seizures, myoclonic seizures, and tonic seizures. The initial seizure types of the patients were partial seizures in 4 cases (patients 3, 5, 6, and 7), tonic seizures in 1 case (patient 2), tonic-clonic seizures in 2 cases (patients 1 and 10) and spasms in 3 cases (patients 4, 8, and 9). One case (patient 3) had an average number of seizures a few times a month, and all were associated with high fever. Three cases (patients 2, 6, and 8) had an average number of seizures of ≤5 times per day, and 6 cases (patients 1, 4, 5, 7, 9, and 10) had an average number of 10–20 seizures per day. Five patients (patients 2, 3, 6, 7, and 8) had a history of seizure with fever, and intracranial infection was eliminated in 4 patients (patients 2, 3, 7, and 8). In patient 6, cerebrospinal fluid examination showed positive MOG antibody. MRI showed abnormal signal near white matter in lateral ventricle triangle. There was still epileptic seizure after glucocorticoid and gamma globulin treatment, and the genetic examination showed *STXBP1* gene mutation.

**Table 3:** Clinical features of patients with STXBP-1 mutations.

Patient number	1	2	3	4	5	6	7	8	9	10
Age at study	2 years 11 months	1 year 2 months	1 year 5 months	3 months 17 days	1 year 10 months	9 months 10 days	8 months 20 days	1 year 1 month	2 years 7 months	1 month 11 days
Age at onset	1 month	1 year 1 month	1 year	2 months 23 days	10 months 25 days	9 months	8 months 14 days	1 year 1 month	1 month	9 days
sex	M	M	M	F	M	M	F	M	F	F
area	urban	urban	rural	rural	rural	rural	rural	rural	urban	rural
Seizure type (s)	tonic-clonic, spasms	tonic-clonic seizure,spasms, absence seizure	fever sensitivity, focal seizure, tonic	spasms, focal seizure	focal seizure, myoclonic	focal seizure	focal seizure	spasms, clonic, focal seizure, myoclonic	spasms, absencetonic-clonic, focal seizure	spasms, absencetonic-clonic, tonic-clonic, focal seizure
EEG at onset	burst suppression	multifocal epileptic activities	slow background, focal epileptic activities	focal epileptic activities, spasms	slow background, focal epileptic activities	slow background, focal epileptic activities	multifocal epileptic activities	slow background	Hypsarrhythmia	burst suppression
MRI	subdural effusion	nomal	nomal	Hypoxic ischemic change	nomal	delayed myelination	delayed myelination	nomal	nomal	nomal
evolution of treatment	PB, NZP + Pred, NZP + TPM	LEV	PB, VPA + CZP	LEV, LEV + TPM, VPA + TPM, VPA + TPM + LEV	OXC + CZP, OXC + CZP + VPA, VPA + LEV, VNS	PB, OXC + TPM, OXC + VPA + CZP + TPM, VNS	OXC, OXC + CZP, CZP + VPA, CZP + VPA + TPM	LEV	TPM, TPM + NZP	NZP + TPM, NZP + TPM + LEV, ACTH, ACTH + vigabatrin, TPM + LEV + vigabatrin
respond to therapy	sz free for near 8 years 8 months	sz free for more than 5 years	sz free for more than 3 years 5 months	sz free for more than 4 years 8 months	sz free for more than 2 years 6 months	sz free for more than 3 years 8 months	sz free for more han 3 years 3 months	sz free for more than 3 years 3 months	sz free for more than 6 years 4 months	no obvious seizures for 2 months
EEG follow up (age)	Hypsarrhythmia (4 months) multifocal and focal epileptic activities (2 years 11 months)	multifocal epileptic activities (4 years 3 months)	nomal (2 years 10 months)	nomal (5 months)	nomal (2 years 9 months)	slow background (2 years 5 months)	multifocal epileptic activities (1 year 10 months)	slow background (3 years 2 months)	Focal epileptic activities (2 years 7 months)	–
Current developmental or intellectual disability	no walk or speech, No social contact (9 years)	poor social contact (4 years 2 months)	nomal (3 years 9 months)	able to walk or speech, poor social contact (5 years)	able to say single syllables.No social contact (4 years)	nomal (4 years 7 months)	able to say single syllables (3 years)	able to say single syllables poor social contact (4 years 5 months)	able to say a few words,poor social contact (7 years)	No walk or speech, No social contact (1 year 6 months)
family history	no	mother	aunt (father's sister)	no	sister	no	no	no	no	No
History of hypoxia	no	no	no	yes	no	no	no	no	no	no
Diagnosis	OS with evolution to IESS	EOEE	EOEE	IESS	EOEE	EOEE	EOEE	EOEE	IESS	OS
Clinical groups	DEE	DEE	GEFS+	DEE	DEE	Focal epilepsy	DEE	Focal epilepsy	DEE	DEE

F, female; M, male; sz, seizure; PB, phenobarbital; NZP, nitrazepam; Pred, prednison; TPM, topiramate; CZP, clonazepam; VPA, valproic acid; OXC, oxcarbazepine; LEV, Levetiracetam; VNS, vagus nerve stimulation; ID, intellectual disability; ACTH, adrenocorticotropic hormone; EEG, electroencephalograms; MRI, magnetic resonance imaging; IESS, Infantile epileptic spasms syndrome; OS, Ohtahara syndrome; EOEE, early-onset epileptic and encephalopathy; DEE, developmental and epileptic encephalopathies; GEFS+, genetic epilepsies with febrile seizures (FS) plus.

Reviewing the above clinical characteristics of all patients, we could distinguish 3 different phenotypic groups. (1) Seven patients (patient 1, 2, 4, 5, 7, 9, 10) had intractable seizures, occurrence of developmental stagnation or regression after seizure onset, and additional neuropsychiatric deficits compatible with developmental and epileptic encephalopathy (DEE). (2) Patient 3 had an average number of seizures a few times a month, and all were associated with high fever. All the times, two seizure types presented during the course of the disease, focal seizures and tonic seizures, and had a relatively benign course, generally good drug response, normal development, and mild neuropsychiatric symptoms, corresponding to genetic epilepsies with febrile seizures plus (GEFS+). (3) The last 2 patients (patient 6, 8) with some form of focal epilepsy.

**Figure 2:**
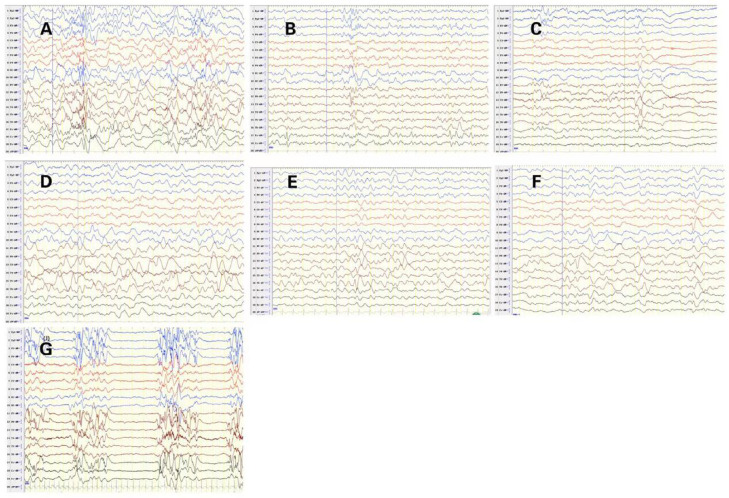
Initial electroencephalograms of patients with syntaxin-binding protein 1 (STXBP1) mutations. (**A**) Patient2 1year and 1months, Multifocal spikes and slow waves during sleep status. (**B**) Patient3 1year and 5months, slow background, multifocal slow waves. (**C**) Patient4 2 months and 29 days, spasms and focal epileptic activities originating from the left Anterior and middle temporal area. (**D**) Patient5 1 year and 2 months, slow background and Slow wave activity originating from the left middle and posterior temporal regions. (**E**) Patient6 9 months and 10days, sharp-slow waves originating from the right Posterior temporal area during sleep status on interictal EEG. (**F**) Patient7 8 months and 20 days, slow and spikes-slow waves originating from bilateral Parietal, Occipital, mid-posterior temporal areas during sleep status. (**G**) Patient10 1 months, burst suppression (BS).

EEG abnormalities were observed in all patients, including burst suppression (patients 1 and 10), hypsarrhythmia (patient 9), multifocal epileptic activities (patients 2, 3, 7), focal epileptic activities (patients 4, 5, and 6), and slow background (patients 3, 5, 6, 8). The initial EEG of 7 patients with *STXBP1* mutations is shown in Figure 2. The other 3 patients had no initial EEG. At the last follow-up, EEG showed normalization after treatment in 3 cases (patients 3, 4, and 5). The initial brain MRI did not present any apparent structural abnormality. Of the nine patients included here, one patient had subdural effusion (patient 1), one had hypoxic-ischemic change (patient 4), and two had delayed myelination (patients 6 and 7). Blood biochemical examination showed no apparent abnormalities.

### Treatment and prognosis

With regard to the treatment and prognosis, including the use of VNS, huge improvements in seizure activity were found in all of our cohorts. With ongoing brain maturation, patient 1, who did not become seizure-free during the first few months of life, developed epileptic spasms. Together with the observation that none of the patients with *STXBP1* mutations evolved to West syndrome later in life after controlling seizure. Four cases (patients 2, 4, 5, and 8) were free of seizures after treatment with LEV. Two patients received LEV monotherapy, they got seizures free when the dose of LEV got 40 mg/kg/d. Others received LEV combination therapy. Initially, patient 4 was treated with bolus doses of topiramate (TPM), nitrazepam (NZP) and VPA, but there was no significant improvement. Next, NZP was replaced with LEV, and the seizures were completely controlled. Patient 5 had failed with bolus doses of oxcarbazepine (OXC), clonazepam (CZP) and VPA. Next, CZP and OXC were replaced with LEV, and the seizures were completely controlled after treatment with LEV and VPA. To make things even better, he tried VNS after being seizure-free and improved his psychomotor ability. Patients 2 and 8 were free of seizures after treatment with LEV alone. Patient 6 had frequent convulsions after a two-day history of fever. The CSF analysis showed an elevated white count of 14 per cubic millimeter. Serum MOG antibodies were requested, and he was started on intravenous immunoglobulin, OXC, TPM, and glucocorticoid (GC). Two weeks later, although his repeat lumbar puncture (LP) was negative and the serum titer of MOG antibodies disappeared, his seizure status had not changed. Next, CZP and VPA were added successively; however, the seizures failed to respond to any medical treatment. Fortunately, he tried VNS, and his seizures promptly ceased after 20 days of VNS therapy. Although the effects of treatment on seizure frequency might be quantifiable, the effect on intellectual disability is not so easily assessed. The good news is that one case (patient 5) experienced improved psychomotor ability after VNS.

## Discussion

*STXBP1* is a member of the evolutionarily conserved Sec1/Munc-18 gene family that plays a central role in vesicular docking and fusion. Splicing mutations, a frameshift mutation, and nonsense mutations can lead to loss-of-function, which is a common mechanism underlying *STXBP1-E* (22). In this study, two novel recurrent missense mutations in *STXBP1* (c.1315A > T, c.751G > C) were detected in three patients with unclassified epileptic encephalopathy (patients 5 and 7). We found that all the patients with novel mutations had similar clinical symptoms: early onset seizures and intellectual disability.

Barcia et al. (15) and Wolking et al. (16) demonstrated that the clinical features in *STXBP1-E* are mostly shared with early onset seizures, a poor prognosis with severe intellectual disability and a high mortality rate and a frequent evolution to infantile spasms (IS). However, in our study, 10 patients eventually became seizure-free, three of the 10 patients were West syndrome and they were reducing the development of West syndrome. We consider this result to be related to prompt and early seizure control. If the notion is that seizures must be controlled as soon as possible to optimize prognosis, then optimal targeted therapies are needed to shorten exposure to repetitive seizures.

EOEE are characterized by recurrent clinical seizures and prominent interictal epileptiform discharges seen during the early infantile period. The underlying genetic cause often results in developmental delay in its own right, with the epileptic encephalopathy further adversely affecting development. Frequent epileptiform activity that impacts adversely on development, typically causing slowing or regression of developmental skills. Similar to the study from Wolking et al. (16), all the patients were also divided into four groups (DEE, GEFS+, GTCS and focal epilepsy) according to their clinical characters. In our study, we noted that developmental delay and epilepsy are characteristic features of *STXBP1*-E, in keeping with previous reports (7, 8). Although developmental delay can be seen at seizure onset, some degree of developmental delay is present prior to the onset of seizures in many patients (patients 1, 3, 4, 5, 6, and 10). Furthermore, in 2009, Hamdan et al. (8) reported *de novo* mutations, p. R388X and c.169 + 1G > A in 2 cases, with intellectual disability and nonsyndromic epilepsy. In 2011, Hamdan et al. (9) reported 1 case with a novel *de novo* truncating mutation, c.1206delT/p.402X, presenting with nonsyndromic intellectual disability deficit and no history of epilepsy. These findings suggest no correlation between seizures and intellectual disability, as described previously (23). *STXBP1* plays an important role in many aspects of neurodevelopment.

The initial EEG was abnormal in all patients. The main EEG finding was (multi)focal abnormality, while burst suppression or hypsarrhythmia was observed in three patients. This type of alteration is much less specific. At the last follow-up, the repeat EEG recordings of 3 cases (patients 3, 4, and 5) were normal. Structural imaging with MRI was normal in most cases, although delayed myelination was present in two patients, subdural effusion was observed in one patient, and hypoxic-ischemic change was observed in one patient.

Treating *STXBP1-E* involves a multidisciplinary approach, including epilepsy control and neurological rehabilitation. Currently, there is no treatment for intellectual, motor or behavioral disturbances, which exert a significant impact on the quality of life of patients. Several studies have reported antiepileptic effects of LEV through modulating the synaptic vesicle release affected by *STXBP1* mutations (11, 24). LEV had a specific effect on the children with *STXBP1-E* in our study, and four patients presented a dramatic response to LEV with full epilepsy control. Among the various antiepileptic drugs, valproic acid and topiramate were partially effective in some patients. Serum MOG antibodies were found in patient 6, and he was given immunomodulation treatment. Although the study from Lopez et al. (25) have identified *STXBP1* as an important player in cytotoxic lymphocytes function, the immunomodulation treatment did not reduce seizure status in patient 6. Further studies are needed to clarify the correlation between *STXBP1-E* and autoimmune mechanism.

VNS has been used in the treatment of epilepsy in clinic, but the mechanism of its application is not completely clear, its mechanism may be: 1. VNS can increase the number of neurotransmitters, such as norepinephrine, 5-hydroxytryptamine and γ-aminobutyric acid (γ-aminobutyricacid) which can produce antiepileptic effect (26, 27). VNS treatment can cause the desynchronization of cortical electrical activity, and the number of spike waves and electrodes decreased significantly during the opening phase (28). The mechanism of VNS in the treatment of epilepsy might be the asynchrony of neural circuits in the hippocampus and thalamic cortex (29). At present, it is believed that the indications for the treatment of epilepsy with VNS include drug refractory epilepsy, unable to take other surgical treatment or poorly controlled epilepsy after other types of surgery. VNS is a relatively safe surgical method, which can be tolerated by most patients, it has low incidence of complications. Because the mechanism of this operation is not clear, ASMs is still the first choice for epileptic control. Fortunately, 1 patient showed improvement in cognitive function after VNS. This improvement may be a direct effect of VNS on behavior, concentration, and affect (30) and may be related to seizure reduction, reduced ASM load in association with successful antiepileptic treatment or putative effects of VNS on mood (26). The most favorable treatment regimens should note the improvement of the prognosis on both seizures and psychomotor ability. VNS may represent the patient's option for optimal seizure and cognitive outcomes. A recent study identified miR-218 and miR-424 as regulators of *STXBP1* expression. Inhibiting their interaction with *STXBP1* resulted in an increase in *STXBP1* protein levels (27). Guiberson et al. identified three chemical chaperones, trehalose, sorbitol, and 4-phenylbutyrate, which are able to restore *STXBP1* protein levels (28) and rescue synaptic deficits. A clinical pilot trial of 4-phenylbutyrate in a small group of *STXBP1* patients began in 2020, and it will be the first trial of a disease-modifying therapy in this patient population (27). Further studies are required to screen for and identify molecules that are effective for both wild-type and mutant *STXBP1*.

In conclusion, *STXBP1* analysis should be considered for infants with seizures and severe ID, and we have shown that the major clinical features of *STXBP1* mutations are frequent seizures from epilepsy, abnormal initial EEG, and intellectual disability. Frequent seizures with fever should also be considered with the *STXBP1* mutation when intracranial infection is eliminated. This study demonstrates better response to LEV in *STXBP1* disorder. Thus, we would suggest early consideration of the use of LEV in this population. VNS may be worthy of consideration as an option for treating *STXBP1*-E. Further studies are needed to determine the adequacy and ideal duration of VNS for optimal management.

## Data Availability

The raw data supporting the conclusions of this article will be made available by the authors, without undue reservation.
