# Supplementary material for: Genotype and phenotype spectrum of 10 children with STXBP1 gene-related encephalopathy and epilepsy
Source: Front Pediatr. 2022 Nov 11;10:1010886. doi: 10.3389/fped.2022.1010886 (PMC9695404; doi:10.3389/fped.2022.1010886)

Figure S1 Conservative analysis of amino acid

Schematic diagram of pathogenic variant sites in nucleic acids and the results of multiple sequence alignment analysis of nine patients: p. R122X, p. E185fs*28, p. P187L, p. R190Q (two patients), p. Q250X, p. A251P, p. I439F, p. R551C.

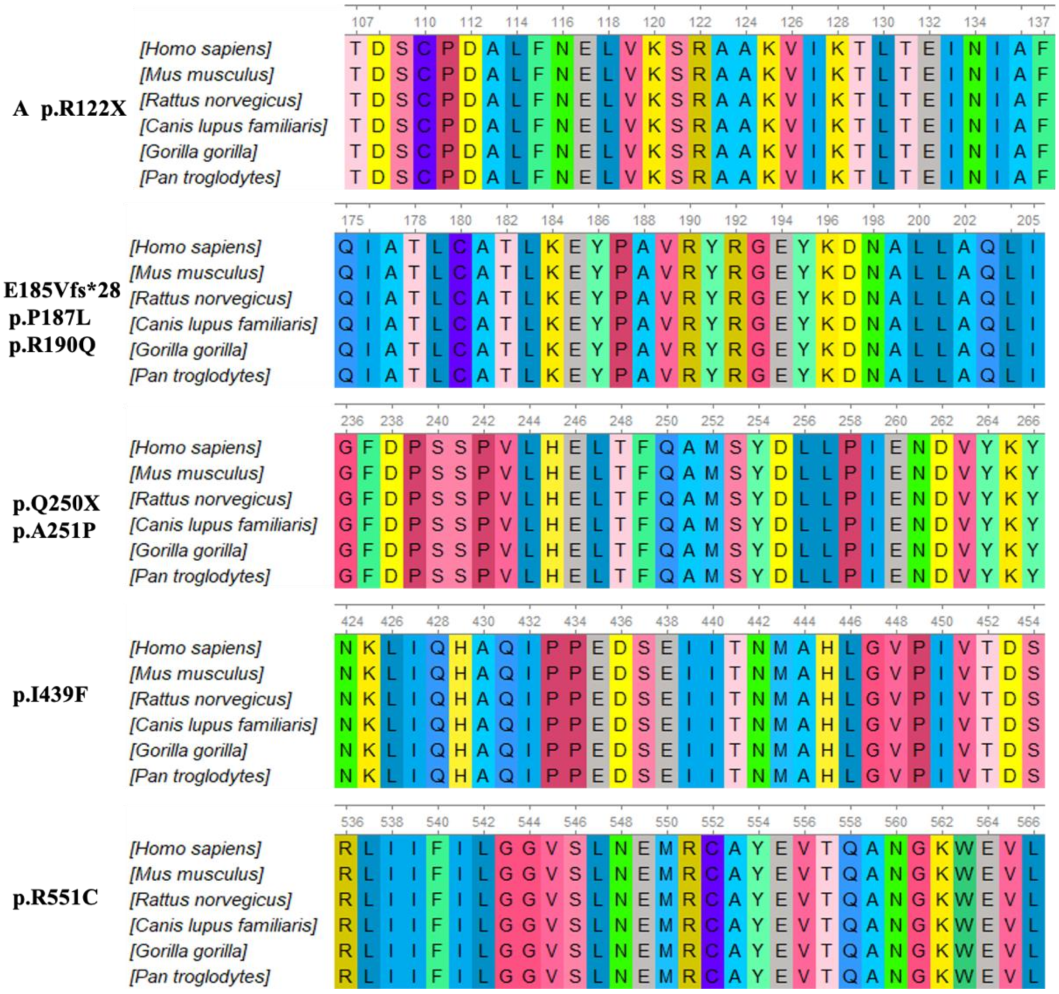

Figure S2 Conservative and in silico analysis. Swiss-model homology modeling software was used to predict protein 3d structure. In the cartoon structure (overall structure), blue represents the α alpha helix, purple represents the β beta fold, and pink coils represent the Loop structure. The spherical structure used at the site of the mutation, the hydrogen bond is a stick structure in which each color represents a different atom. Among them, yellow represents C atoms, gray represents H atoms, blue represents N atoms, red represents O atoms, and orange represents S atoms. (A) the structure prediction of frameshift mutation (c.554_559delinsTGTG, p. E185fs*28). (B) the structure prediction of wild-type STXBP1 protein. (C) the structure prediction of missense variant. (D) the partial enlargement of missense variant (c.1315A>T, p.I439F). (E) the partial enlargement of wild-type STXBP1 protein. (F) the partial enlargement of missense variant (c.751G>C, p.A251P). (G) the partial enlargement of wild-type STXBP1 protein.

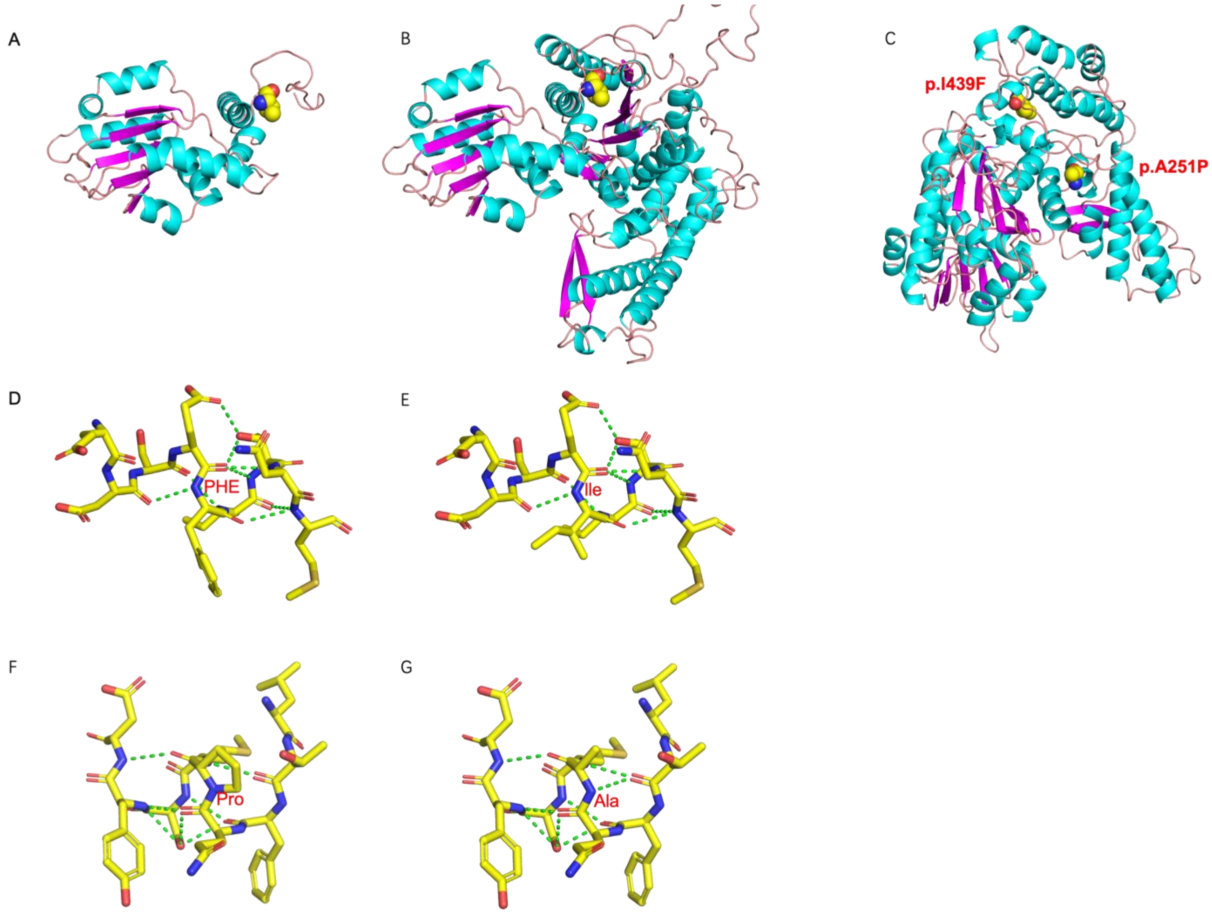

Supplement: Supplementary file 1 [file Datasheet1.pdf]
